# An fMRI Study of the Neural Systems Involved in Visually Cued Auditory Top-Down Spatial and Temporal Attention

**DOI:** 10.1371/journal.pone.0049948

**Published:** 2012-11-15

**Authors:** Chunlin Li, Kewei Chen, Hongbin Han, Dehua Chui, Jinglong Wu

**Affiliations:** 1 Biomedical Engineering Laboratory, Graduate School of Natural Science and Technology, Okayama University, Okayama, Japan; 2 School of Biomedical Engineering, Capital Medical University, You An Men, Beijing, China; 3 Banner Alzheimer’s Institute, Department of Psychiatry, Phoenix, Arizona, United States of America; 4 Department of Neurology, Peking University Third Hospital, Beijing, China; 5 Neuroscience Research Institute and Department of Neurobiology, Key Laboratory for Neuroscience, Ministry of Education and Ministry of Public Health, Health Science Center, Peking University, Beijing, China; Baycrest Hospital, Canada

## Abstract

Top-down attention to spatial and temporal cues has been thoroughly studied in the visual domain. However, because the neural systems that are important for auditory top-down temporal attention (i.e., attention based on time interval cues) remain undefined, the differences in brain activity between directed attention to auditory spatial location (compared with time intervals) are unclear. Using fMRI (magnetic resonance imaging), we measured the activations caused by cue-target paradigms by inducing the visual cueing of attention to an auditory target within a spatial or temporal domain. Imaging results showed that the dorsal frontoparietal network (dFPN), which consists of the bilateral intraparietal sulcus and the frontal eye field, responded to spatial orienting of attention, but activity was absent in the bilateral frontal eye field (FEF) during temporal orienting of attention. Furthermore, the fMRI results indicated that activity in the right ventrolateral prefrontal cortex (VLPFC) was significantly stronger during spatial orienting of attention than during temporal orienting of attention, while the DLPFC showed no significant differences between the two processes. We conclude that the bilateral dFPN and the right VLPFC contribute to auditory spatial orienting of attention. Furthermore, specific activations related to temporal cognition were confirmed within the superior occipital gyrus, tegmentum, motor area, thalamus and putamen.

## Introduction

The widely used experimental paradigm used to study orienting of visual spatial attention was first developed by M.I. Posner [1]. In this paradigm, a spatial cue (usually an arrow) is presented in the center of the visual field (pointing either left or right), providing a spatial hint of the location of an upcoming target stimulus. Using this information, participants can predict the location of a target and voluntarily pay attention to that location. This voluntary visual spatial attention is usually referred to as visual top-down spatial attention.

Using the Posner paradigm to investigate top-down spatial attention, fMRI studies have revealed the importance of the frontoparietal network (FPN) in these processes [2], [3], [4]. This dorsal FPN consists of the bilateral inferior parietal sulcus (IPS) and the frontal eye field (FEF). Furthermore, right hemisphere dominance has been reported during top-down visual spatial attention tasks involving the inferior parietal lobe/temporal parietal junction (IPL/TPJ), the dorsolateral prefrontal cortex (DLPFC) and the ventrolateral prefrontal cortex (VLPFC) [4].

Similar experiments can also be used to study temporal (as opposed to spatial) attention. For instance, in one experiment, two concentric circles (rather than an arrow) were presented in the center of the field of vision, which is a commonly used visual temporal cue for determining the neural correlates of visual top-down temporal attention. The circles provide a hint of time interval (short or long) between the cue and target stimuli [2], [5]. Specifically, when the inner circle was presented, it meant the target stimuli would be presented after a short interval; when the outer circle was presented, it meant the target stimuli would be presented after a longer interval. According to the imaging result, the same FPN network involved in spatial attention was also involved in visual top-down temporal attention [2], [5].

Recently, the Posner paradigm (i.e., a visual spatial cue followed by a subsequent target stimulus) was used to study auditory top-down attention. Smith et al. [6] investigated the neural networks involved in visual and auditory top-down spatial attention and found similarities in the neural activation during both processes. In their study, a visual spatial cue was used, followed by either a visual target or an auditory target. They concluded that the dorsal FPN (dFPN) also modulated auditory top-down spatial attention (as well as visual top-down spatial attention). This auditory top-down spatial attention related dFPN is attributed to the dorsal part of auditory dual-pathway model [7]. Furthermore, this dFPN relates to auditory spatial cognition [8]. Especially the inferior parietal lobe (IPL) as a role of auditory spatial working memory contributes to the auditory spatial attention [9]. Moreover, involvement of the middle temporal gyrus (MTG) in auditory top-down spatial attention has also been described elsewhere [10].

These previous studies demonstrate the neural correlates involved in visual top-down spatial and temporal attention. Furthermore, studies on the neural correlates of auditory top-down attention have also been well studied by fMRI within the spatial domain. Several studies on auditory temporal attention by using scalp-recorded event-related potentials had shown that pay attention to a special time interval could modulate early perceptual processing [11], affect target detection [12] and facilitate short-term consolidation during a rapid serial auditory presentation task [13], [14]. Moreover, an fMRI study found that, during an auditory time estimation task (duration discrimination of tone pairs in the range of 1,000–1,400 ms), including the right medial and both left and right dorsolateral prefrontal cortices (DLPFC), thalamus, basal ganglia (caudate nucleus and putamen), left anterior cingulate cortex (CC), and superior temporal auditory areas were activated [15]. However, the neural systems that are important for auditory top-down temporal attention still remain unclear. Furthermore,differences between the mechanisms of top-down spatial and temporal attention process are still unclear.

To determine the neural correlates involved in auditory top-down temporal attention and to compare them with the neural correlates involved in auditory top-down spatial attention, the current study was designed to investigate the areas of the brain activated by auditory targets following a spatial or temporal visual pre-cues, respectively. The process of neutral attention (i.e., an auditory target following a visual neutral cue) was observed to determine nonspecific attention-related activity and used to cancel out basic visual and auditory cognition mediated activity. We confirm that increased activation was observed during spatial tasks within the dorsal FPN, but not during the temporal tasks, due to the absence of FEF activity. Activation of the rVLPFC was significant during spatial attention tasks than during temporal or neutral attention tasks. The activation of the DLPFC was not observed to be different between the three attention tasks. Specific activations related to temporal cognition were confirmed within the superior occipital gyrus, tegmentum, motor area, thalamus and putamen.

## Results

### Behavioral Results

**Figure 1:**
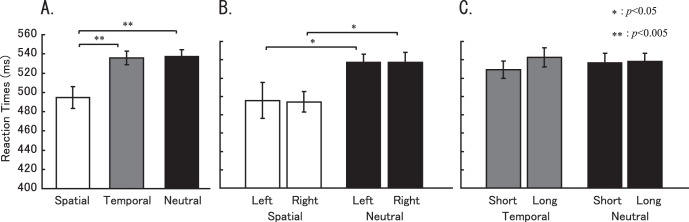
RTs for each attentional tasks.

Behavioral data were derived from the participants’ performances during the fMRI experiments. Approximately 80%–97% response accuracy was observed for all the subjects (except one participant, who was 78% accurate). Figure 1 shows the averaged RTs for tasks. RTs during the spatial attention task, temporal attention task, neutral task and control task are shown in Figure 1A and summarized in Table 1. Using Bonferroni correction (at *p*<0.05) to test the pair-wise comparisons, post hoc analyses indicate the RTs during the spatial attention task that were different from those during the temporal attention task (*p*<0.003) and the neutral attention task (*p*<0.002). No differences were found between the temporal and neutral attention tasks (*p* = NS). We also divided the target appearance into left or right, short or long for observing the differences between tasks. We found that there were significant differences when compared left targets during Spatial and Neutral task (*p*<0.05), and also right targets during Spatial and Neutral task (*p*<0.05). However, we did not find any significant difference between tasks when compared short or long interval.

**Table 1:** Average reaction times (RTs) for each task.

Task	Condition	ReactionTime (ms)	StandardError (SE)
Spatial	Total	494.8	11.4
	Left target	495.5	19.7
	Right target	494	11.1
Temporal	Total	536	7
	Short interval	529.3	9.3
	Long interval	542.7	10.5
Neutral	Total	537.2	7.2
	Left target	537.3	9
	Right target	537.2	11.1
	Short interval	536.9	10.4
	Long interval	538.1	9.2
Control	Total	479.6	32.9

### fMRI Results

Next, we were interested in investigating the areas of activation during auditory target detection with respect to the three attention cues and to investigate how these cues modulate similarities and differences.

### Activation during the Three Attention Tasks

**Figure 2:**
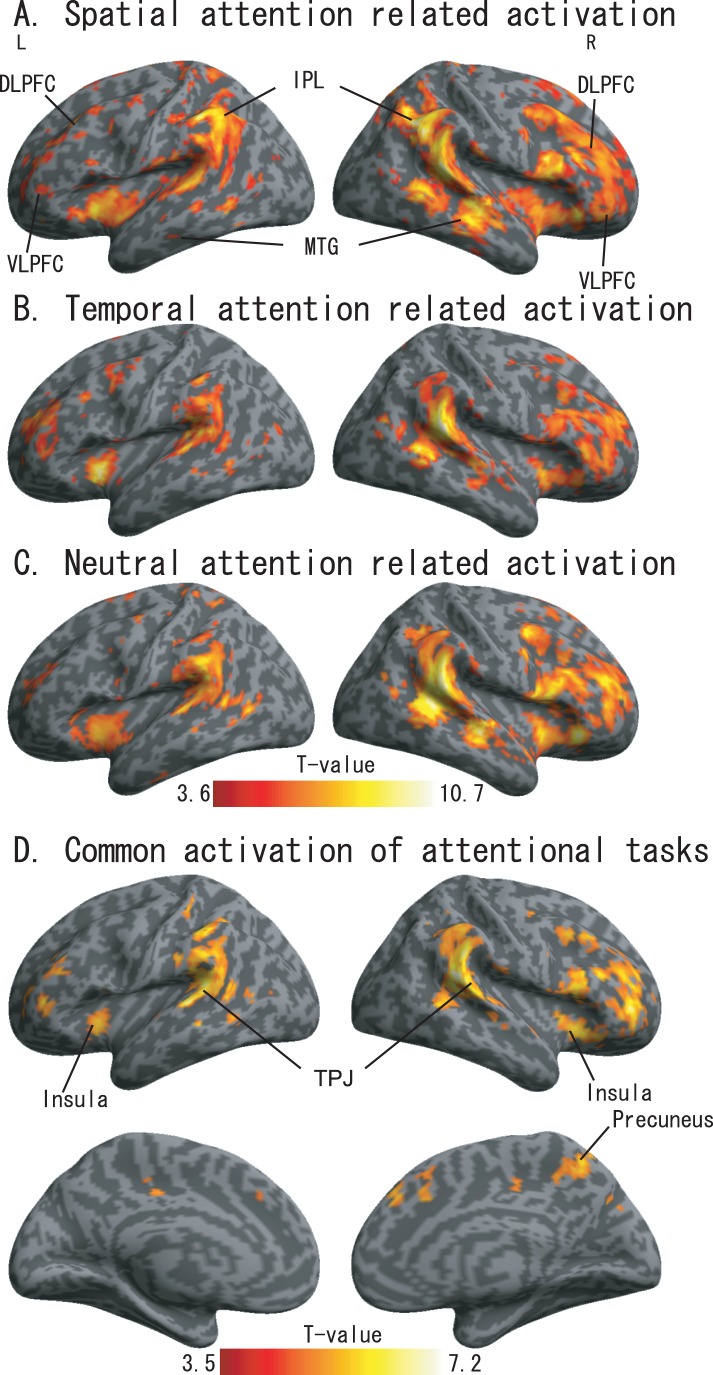
SPM results from the spatial attention task vs. control task (A), temporal attention task vs. control task (B), neutral task vs. control task (C), and common activations of attentional tasks (D). Significance was uncorrected *p*<0.0005.

The activations during the spatial, temporal and neutral tasks are shown in Figure 2A–C and summarized in Table 2. The rendered activation map in Figure 2A–C was generated using an uncorrected threshold of *p*<0.0005 The bilateral IPL/TPJ was activated under each of the 3 conditions, and other similarities were observed between the three tasks. Activation of the bilateral insular (BA13/38) and the MFG (BA6/8/9), along with activation of the TPJ. We also analyzed the commonly activated regions between the three attention tasks using conjunction contrast in the one-way ANOVA analyses. The rendered activation map is shown in Figure 2D under a threshold of uncorrected *p*<0.0005. By comparing the activations among the three tasks, we noted that the bilateral superior temporal gyrus (STG, BA41/42) was activated across all tasks, as was the frontal cortex, which included the anterior insula (BA13/38), the VLPFC (BA44/45/47) and the DLPFC (BA9/10/46). Activation of the precuneus (BA5/7) was observed across the three tasks.

**Table 2:** Activations related to attentional conditions.

Region	Hemisphere	cluster size (voxels)	Brodmann area	Coordinate (x, y, z, mm)	z-score
Spatial vs. Control					
IPL	R	32537	40	50 −54 42	5.62
IPL	L		40	−56 −42 42	5.61
MFG	R		46	46 44 20	5.6
Insula	L			−38 10 −2	4.64
MFG	L		8	−32 28 40	4.37
CC	R		24	6 −22 42	4.18
Precuneus	R	2359	7	10 −62 64	5.08
MFG	L	198	4/6	−8 −4 64	4.73
Cerebellum	R	389		16 −48 −28	3.84
Temporal vs. Control					
IPL	R	2489	22	60 −40 16	5.23
IFG	R	3897	47	44 18 2	4.22
IPL	L	2921	40	−62 −52 38	4.32
IFG	L	482	47	−34 44 6	4.12
Insula	L	318		−32 12 −4	4.65
Precuneus	R	259	7	10 −54 56	3.98
ACC	R	158	9	4 22 44	3.89
Neutral vs. Control					
IPL	R	15230	40	58 −40 20	5.82
IPL	L	12652	40	−56 −28 20	5.75
ACC	R	2511		8 22 36	5.12
SFG	L	259	8	−40 −2 58	4.44
PCC	R	358		10 −72 34	4.12
Cerebellum	R	193		12 −52 −16	3.99

Significance was set at an uncorrected threshold of *p*<0.0005 and K = 150 voxels. The approximate anatomical regions and Brodmann areas are shown according to the Talairach atlas, and the x, y, and z coordinates are from the SPM5. Abbreviations are as follows: SPL - Superior Parietal Lobe, IPL - Inferior Parietal Lobe, STG - Superior temporal Gyrus, MTG - Middle temporal Gyrus, SFG - Superior Frontal Gyrus, IFG - Inferior Frontal Gyrus, CC - Cingulate Cortex, PFG - Prefrontal Gyrus, MFG - Middle Frontal Gyrus, R - right hemisphere, L - left hemisphere, and M - bilateral hemisphere.

### Main Effects of the Attention Tasks

**Figure 3:**
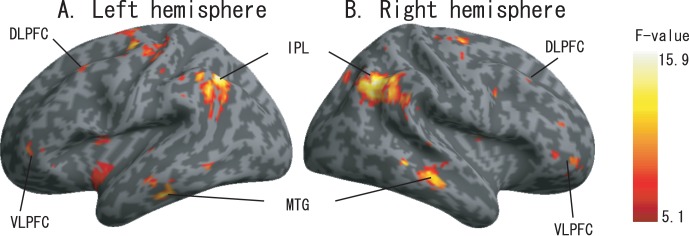
The main effects of auditory attention results. The activation maps are showing the differences in activation between the three conditions (spatial vs temporal vs neutral, displayed at p<0.01 uncorrected,). A. Left hemisphere. B. Right hemisphere.

We note in particular that the bilateral DLPFC and the VLPFC showed activations across all three of the attention tasks, although the magnitude of activation varied (Figure 3). Figure 3A and 3B) show the rendered activation differences among the three attention tasks (using uncorrected *p*<0.01 for illustration purposes). The bilateral DLPFC, the VLPFC, the IPL and the MTG as well as the bilateral FEF showed significant differences between the 3 conditions. Pair-wise comparisons were performed on the extracted data. Significant differences were found in regions of the right VLPFC between spatial and temporal attention tasks (*p* = 0.001), and spatial and neutral attention tasks (*p* = 0.01); in the right IPL between spatial and temporal attention tasks (*p* = 0.005), spatial and neutral attention tasks (*p* = 0.005); in the right MTG between spatial and temporal attention tasks (*p* = 0.05); in the left IPL between spatial and temporal attention tasks (*p* = 0.05), and spatial and neutral attention tasks (*p* = 0.01); and in the left MTG between spatial and temporal attention tasks (*p* = 0.05). Interestingly, the blood oxygen level-dependent (BOLD) signal changes observed during the spatial attention tasks were higher than those during the temporal and neutral attention tasks in all the regional of interesting (ROI) results.

### Significant Activations Observed between Spatial Tasks vs. Temporal Tasks, and Spatial Tasks vs. Neutral Tasks

**Figure 4:**
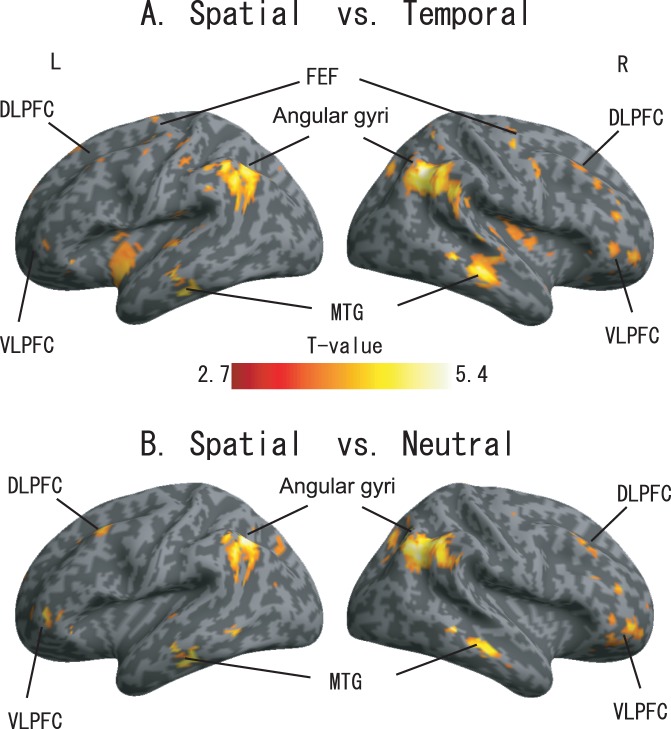
Activation difference between (A) spatial task and temporal task (B) spatial task and neutral task. Significant activations at an uncorrected threshold of *p*<0.005.

To further confirm the differences observed across the conditions, the activations were compared between (A) spatial and temporal tasks (spatial task – temporal task, in brain regions where the activations under spatial attention were significant, *p*<0.05 uncorrected) and (B) spatial and neutral tasks (spatial task – neutral task, in brain regions with significant spatial attention activations, *p*<0.05 uncorrected). The rendered results are shown in Figure 4A and 4B, and the rendered activation is displayed under a threshold of uncorrected *p*<0.005. Figure 3I and 3J show the ROI-based BOLD signal changes within the left FEF (x = −28, y = −16, z = 56 with an 8 mm radius) and the right FEF (x = 28, y = −14, z = 54 with an 8 mm radius), respectively.

**Figure 5:**
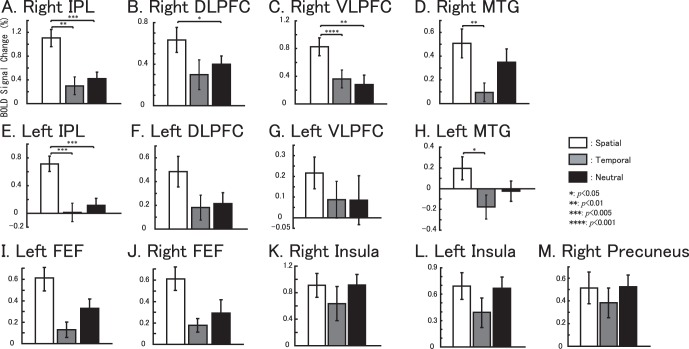
Anatomical ROI-based BOLD signal changes for the three attentional tasks are shown in (A) Right IPL, (B) Right DLPFC, (C) Right VLPFC, (D) Right MTG, (E) Left IPL, (F) Left DLPFC, (G) Left VLPFC, (H) Left MTG, (I) Left FEF, (J) Right FEF, (K) Right insula, (L) Left insula and (M) Right precuneus. White bars represents the spatial task vs. control task, gray bars represents the temporal task vs. control task and black bars represents neutral task vs. control task. The error bar represents the standard error (SE). Non-parametric comparisons results are shown in the bars (*: *p*<0.05, **: *p*<0.01, ***: *p*<0.005, ****: *p*<0.001).

Figure 5A–H shows the BOLD signal-change results based on anatomical ROIs (data were extracted from left the DLPFC, the right DLPFC, the left VLPFC, and the right VLPFC, respectively) together with the ROI-based one-way ANOVA results (summarized activations are shown in Table 3). The ROI-based BOLD signal changes within the right precuneus (x = 12, y = −52, z = 50 with an 8 mm radius), the right Insula (x = 42, y = 22, z = −14 with an 8 mm radius) and the left insula (x = −32, y = 18, z = −6 with an 8 mm radius) did not show significant difference among the tasks (Figure 5K–M). Figure 5I and 5J demonstrate that the bilateral FEF was not activated during temporal attention tasks, and the highest BOLD signal changes were observed during the spatial and neutral tasks within the right FEF.

**Table 3:** Activations analyzed by the main effects of auditory attention.

Region	Hemisphere	cluster size (voxels)	Brodmann area	coordinate (x, y, z, mm)	z-score
Supramarginal	L	733	39/40	−42 −52 36	4.38
MTG	R	390	1/21	62 −34 −8	4.36
Supramarginal	R	1270	39/40	50 −56 40	4.28
MTG	L	204	20/21	−48 −26 −18	4.08
Precentral Gyrus	L	391	4/6	−28 −16 56	3.82
Precentral Gyrus	R	102	6	28 −14 54	3.74
SFG	L	151	6	−18 −12 70	3.28
MFG	L	159	9	−36 22 52	3.2
Culmen	R	40		10 −48 −14	3.19
IFG	R	25	47	32 34 −22	3.14
SFG	R	79	10	34 58 −4	3.13
CC	L	215	24	−2 −2 46	3.13
MFG	R	31	11	28 46 −8	3.13
MFG	M	162	8	14 34 62	3.06
IFG	L	53	47	−38 46 2	3.03
MFG	R	35	45	44 44 20	3.01
MFG	R	38	9	42 28 42	2.99
MFG	R	45	6	30 6 40	2.96
Extra-Nulear	L	125		−34 2 −10	2.95
SFG	R	54	6	36 −12 68	2.94
SFG	R	85	6	8 −20 78	2.93
SFG	R	42	9	10 46 44	2.92
Extra-Nulear	R	42		36 −2 18	2.9
Declive	L	37		−6 −62 −18	2.8
Extra-Nulear	L	38		−30 2 10	2.78
IFG	R	28	47	42 42 0	2.69
IPL	L	27	40	−58 −40 36	2.65
MFG	L	56	9	−4 40 28	2.61

Significant activations at an uncorrected threshold of p<0.01. The approximate anatomical regions and Brodmann areas are from the Talairach atlas, and the x, y, and z coordinates are from the SPM5. Abbreviations are as follows: SPL - Superior Parietal Lobe, IPL - Inferior Parietal Lobe, STG - Superior Temporal Gyrus, MTG - Middle Temporal Gyrus, SFG - Superior Frontal Gyrus, IFG - Inferior Frontal Gyrus, CC - Cingulate Cortex, PFG - Prefrontal Gyrus, MFG - Middle Frontal Gyrus, R - right hemisphere, L - left hemisphere, and M - bilateral hemisphere.

### SVC Analysis Results Based on Anatomical Regions

Anatomical region-based small volume correction (SVC) results are summarized in Table 4. The SVC results indicated that the MTG, IPS and prefrontal cortex (PFC) were activated significantly more during spatial tasks compared with temporal and neutral tasks. Specifically, the MTG was activated during all the three tasks, but it was activated much more significantly during spatial tasks compared with temporal or neutral tasks (BA21/22). Significant differences were also observed in the right IPL/TPJ (spatial vs. temporal) and the left IPL/TPJ [16] (spatial vs. neutral). It is important to note that, among the four anatomical regions within the PFC, significant differences were observed only between the spatial and temporal tasks and between the spatial and neutral tasks within the right VLPFC.

**Table 4:** Small Volume Correction (SVC) results based on anatomical masks.

		Spatial vs. Temporal		Spatial vs. Neutral	
		Coodinate	P value	Coodinate	P value
	Region	(x, y, z, mm)	(family-wise error)	(x, y, z, mm)	(family-wise error)
1	Right VLPFC	32 56 −4	*p*<0.029	36 54 −4	*p*<0.025
				28 48 −10	*p*<0.038
2	Right DLPFC				
3	Left VLPFC				
4	Left DLPFC				
5	Right IPS	48 −56 40	*p*<0.001	46 −62 40	*p*<0.001
		42 −52 32	*p*<0.009	42 −58 32	*p*<0.003
				50 −56 40	*p*<0.004
				48 −50 34	*p*<0.007
				38 −64 44	*p*<0.009
6	Left IPS	−42 −50 34	*p*<0.002	−46 −56 40	*p*<0.001
		−48 −60 36	*p*<0.005	−42 −52 34	*p*<0.001
7	Right MTG	62 −30 −10	*p*<0.004	64 −36 −8	*p*<0.001
		64 −26 −10	*p*<0.004	64 −26 −1	*p*<0.011
		64 −36 −8	*p*<0.005	64 −22 −14	*p*<0.014
				68 −26 −12	*p*<0.026
				60 −26 −12	*p*<0.038
8	Left MTG			−62 −26 −18	*p*<0.032
9	Right SC	6 −30 −12	p<0.013		
10	Left SC				

The x, y, and z coordinates are from the SPM5. The approximate anatomical regions are from Talairach atlas, and the x, y, and z coordinates are from the SPM5. SPL - Superior Parietal Lobe, IPL - Inferior Parietal Lobe, STG - Superior Temporal Gyrus, MTG - Middle Temporal Gyrus, SFG - Superior Frontal Gyrus, IFG - Inferior Frontal Gyrus, CC - Cingulate Cortex, PFG - Prefrontal Gyrus, MFG - Middle Frontal Gyrus, SC - Superior Colliculus, R - right hemisphere, L - left hemisphere, and M - bilateral hemisphere.

### Specific Activations Observed during the Temporal and Neutral Tasks

**Figure 6:**
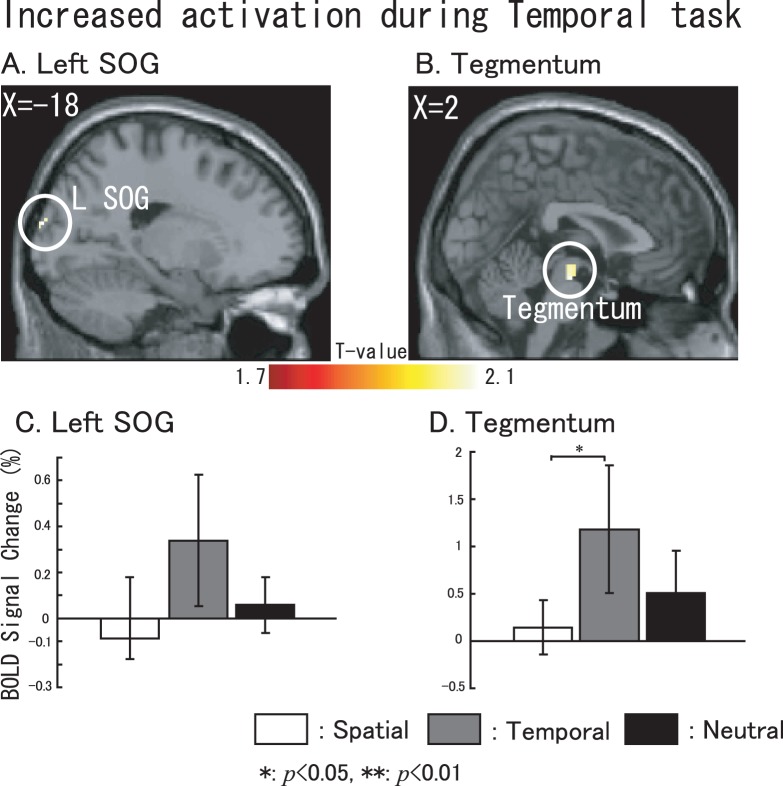
Specific activations involved in temporal tasks. Sliced section results are shown at a threshold of uncorrected *p*<0.05. (A) left SOG. (B) tegmentum within midbrain. BOLD signal changes based on the extracted data from (C) left SOG, and (D) the tegmentum with in midbrain. For C and D, white represents spatial task vs. control task, gray represents temporal task vs. control task and black represents neutral task vs. control task. The error bar represents the standard error (SE). Non-parametric comparisons results are shown in the bar graphs (*: *p*<0.05, **: *p*<0.01).

**Figure 7:**
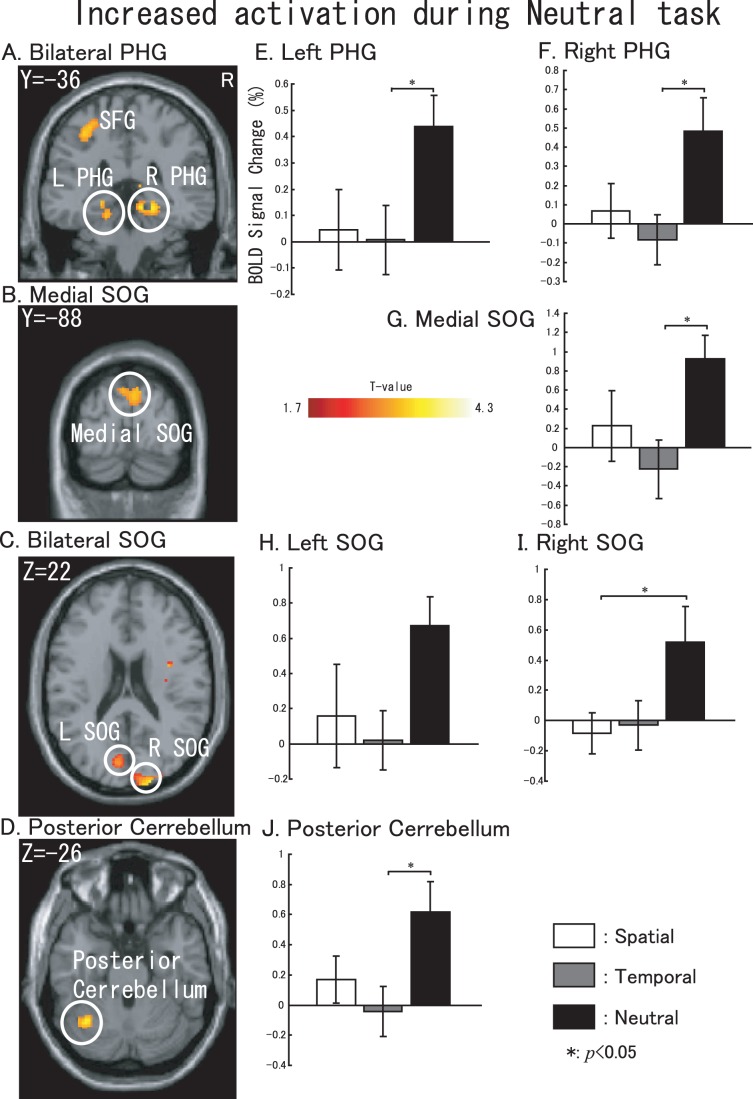
Specific activations involved in neutral task. Sliced section results are shown at a threshold of uncorrected *p*<0.05. (A) left SFG. (B) medial SOG. (C) bilateral SOG. (D) posterior cerebellum. BOLD signal changes based on the extracted data from the (E) left PHG, (F) right PHG, (G) medial SOG, (H) left SOG (I) right SOG, and (J) posterior cerebellum. For E–J, white represents spatial task vs. control task, gray represents temporal task vs. control task and black represents neutral task vs. control task. The error bar represents the standard error (SE). Non-parametric comparisons results are shown in the bar graphs (*: *p*<0.05).

To investigate the particular activations found during temporal attention tasks (and also during neutral attention tasks), we examined the significant activations under temporal or neutral tasks compared with the other two tasks. Contrasts for significant activations during temporal tasks were used for (temporal-spatial)+(temporal-neutral) datasets, and neutral tasks used (neutral-spatial)+(neutral-temporal) datasets. The temporal results are shown in Figure 6, and the neutral results are shown in Figure 7 (*p*<0.05, uncorrected). ROI-based BOLD signal changes were calculated as described below. The left superior occipital gyrus (SOG, x = −18, y = −98, z = 18, cluster size: 22 voxels, with an 8-mm radius) did not show significant differences between the tasks, but the tegmentum in the middle brain (cluster size: 40 voxels, x = 2, y = −18, z = 18, with an 8-mm radius) was particularly activated during the temporal attention process (showed in Figure 6C, 6D). SOG and Tegmentum are the only structures that showed significant when compared temporal to non-temporal tasks. During neutral tasks, several regions including the bilateral parahippocampal gyrus (PHG, Figure 7A, x = −18, y = −36, z = −12, with an 8-mm radius and x = 24, y = −36, z = −8, with an 8 mm radius), the medial SOG (Figure 7B, x = 0, y = −84, z = 36, with an 8-mm radius), the bilateral SOG (Figure 7C, x = −7, y = −79, z = 20, with an 8 mm radius and x = 20, y = −96, z = 20, with an 8-mm radius) and the posterior cerebellum (Figure 7D, x = −34, y = −60, z = −26, with an 8-mm radius) also showed changes. The BOLD signal changes shown in Figure 6E–J correspond to the sliced results.

### Hazard Function Related Activations

**Figure 8:**
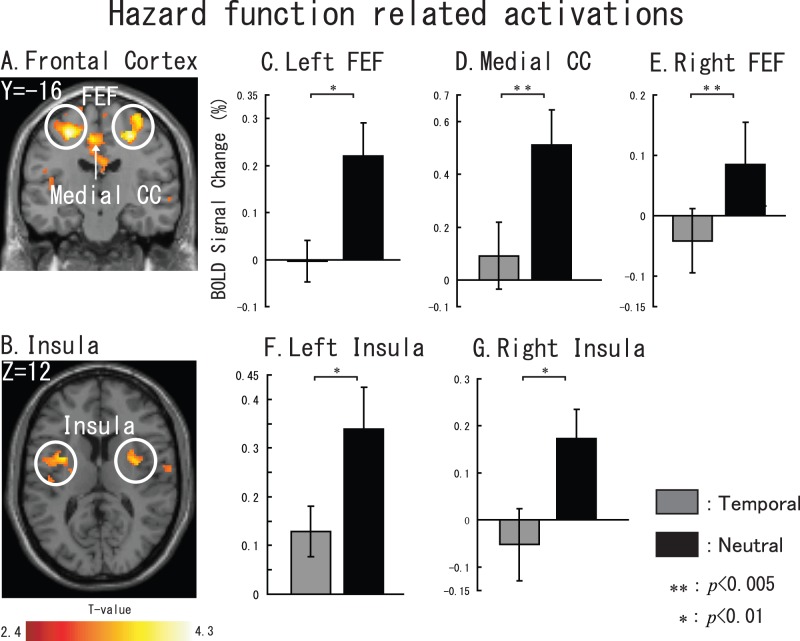
Hazard function related brain activations. Sliced section results are shown at a threshold of uncorrected *p*<0.01. (A) left FEF, medial CC and right FEF. (B) left and right insula. For C–G, gray represents temporal task vs. control task and black represents neutral task vs. control task. The error bar represents the standard error (SE). Non-parametric comparisons results are shown in the bar graphs (*: *p*<0.01, **: p<0.005).

We elicited hazard function related activations by contrast Neutral task-Temporal task. Figure 8 shows the sliced results. We found five brain regions: left FEF (cluster size: 1692 voxels, X = −26, Y = −18, Z = 54), right FEF (cluster size: 404 voxels, x = 28, y = −14, z = 54), medial CC (cluster size: 806 voxels, x = −2, y = −2, z = 46), left insula (cluster size: 1065 voxels, x = −30, y = 2, z = 10), and right insula (cluster size: 780 voxels, x = 36, y = −2, z = 16).

**Figure 9:**
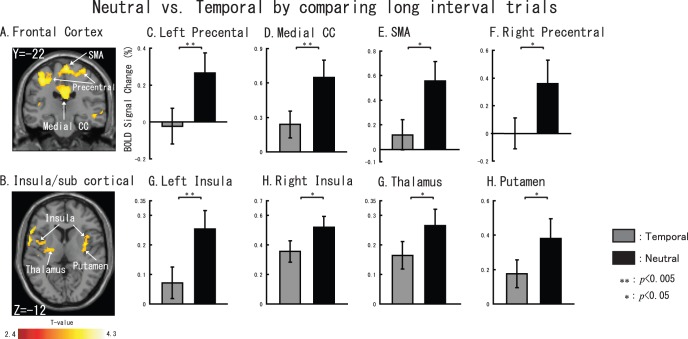
Neutral task vs. Temporal task by comparing long interval trials. Sliced section results are shown at a threshold of uncorrected *p*<0.01. (A) left Prefrontal cortex, medial CC, SMA and right Prefrontal cortex. (B) bilateral insula, thalamus and putamen. For C–H, gray represents temporal task vs. control task and black represents neutral task vs. control task. The error bar represents the standard error (SE). Non-parametric comparisons results are shown in the bar graphs (*: *p*<0.05, **: p<0.005).

Furthermore, with regard to the long interval target cognition during the neutral would cause more ‘temporal effort’, we made a contrast of Neutral task vs. Temporal task only by long interval trials to find out some activations that also related to temporal cognition. Activations are shown in Figure 9. A large activation cluster was found in the motor area and cingulate cortex (cluster size is 4372 voxels) including Bilateral precentral cortex (right: x = 34,y = −22,z = 66; left: x = −34,y = −22,z = 52 ), supplemental motor area (SMA, x = 2,y = −22,z = 68), Medial CC (x = 2,y = −22,z = 34 ) when used a threshold of *p*<0.01 at voxel level. In addition, we also found that bilateral insula (right: x = 42,y = −10,z = 6, cluster size = 533 voxels; left: x = −40,y = 0,z = 10, cluster size = 950 ) and right putamen (x = 38,y = −4,z = 6,) and thalamus (x = −24,y = −12,z = 10, cluster size = 217).

## Discussion

In the present study, we used a cue-target paradigm commonly used in top-down attention studies, and we report on reliability of the whole activations associated with auditory tasks. Keeping in mind the few reports concerning the role of the rVLPFC in modulating auditory attention mechanisms (in both the spatial and temporal domains), we also examined its function in the current study. Our findings help to clarify the role of the rVLPFC, as well as the involvement of a number of other previously reported brain regions, in top-down spatial attention. Furthermore, we reported the particular activations during the temporal task compared to non-temporal attentional tasks as well as comparing the neutral task to specific spatial or temporal tasks.

### Different Behavioral Performance during Attention Tasks

We found that, both left target and right target condition, RTs in spatial task were significantly different from neutral task (left: *p*<0.05; right: *p*<0.05) (Figure 1B). However, both target appeared after a short interval or long interval did not show significant between temporal task and neutral task (Figure 1C). Moreover, RTs to target after a short interval did not show a significant when compared to which after a long interval during the temporal task and neutral task (Figure 1C). We believe this result can be explained by the “hazard function” [17]; during the neutral trials, when a target does not appear after the short delay, it must (by process of elimination) appear after the longer delay, thus explaining why the RTs were not significantly different between temporal and neutral tasks.

### Contributions of dFPN and MTG to Spatial Attention

Fritz et al. [18] and Smith et al. [6] investigated auditory spatial attention using a visual pre-cue and showed activation of the dFPN, including the bilateral FEF and the bilateral IPL. Our findings (Figure 2A.) show consistent activation in the bilateral hemisphere, which has been previously reported in visual spatial attention studies [2], [17]. Within the dFPN, previous studies found IPL participation during the spatial attention process not only in top-down but also in bottom-up unimodal [19], [20] and multimodal attention studies [21], [22], [23]. Moreover, our results demonstrate significantly higher levels of activation during spatial attention tasks compared with temporal and neutral attention tasks. Our findings and those of others, all point to the importance of the IPL in spatial cognition [6], [18]. This dFPN network configuration is attributed to an dual-pathway model in humans as suggested by Alain and his colleagues [7]. Furthermore, Alain et al. also found that the dFPN was employed with sound localization [8], and the IPL contributed as a role of auditory spatial working memory [9].

Another parietal region, the precuneus (BA5/7), was also observed to be activated during all three tasks. These findings are in good agreement with recent fMRI studies that reported precuneus (BA5/7) involvement in shifting not only between visual and auditory attention [24] but also between the position of left and right auditory target stimuli [25].We also found that the right medial temporal cortex (BA21/22) was activated significantly during the spatial and neutral attention tasks, an area which has been associated with selectivity of auditory stimuli involved in spatial or feature aspects [10], [26], [27]. Furthermore, when compared with results from the temporal attention tasks, the left MTG (BA21/22/37) and the right MTG showed more significant activations during the spatial attention tasks. Our results also showed significant activation of the right superior colliculus (SC) during spatial tasks (see SVC results in Table 4), a finding that is consistent with a previous study on primate covert spatial attention [28]. This finding indicates that the SC is not only part of a brain network that directs saccadic eye movements (overtly shifting both gaze and attention from position to position in space) but also contributes to the control of covert spatial attention (a process that focuses attention on a region of space different from the point of gaze). In our study, however, activation differences within the right SC were not observed between spatial and neutral tasks. One possible cause of this insignificance was the fact that the neutral task included both spatial attention and temporal attention processes.

### Spatial Attention Activation within the Right VLPFC

With regard to right VLPFC functions, one fMRI report showed that its activation with right hemisphere dominance was correlated to target detection with careful spatial selection [4]. This finding implies that the right VLPFC is potentially involved in target detection, at least when completed under its important role in the endogenous maintenance of alert state [29], [30]. Another event-related potentials experiment [31] suggested the involvement of the right VLPFC in spatial attention when a visual cue was used for orienting spatial attention to auditory targets. Furthermore, an intracranial electroencephalographic study concluded that the VLPFC encodes spatial information as well as object processing [32].

The right VLPFC was another area of focus in the current study. The results (Figure 2A–C) showed significant activations for all the three attention tasks within the right VLPFC, which were at least partially due to the target detection processes discussed in a previous study [4], and are consistent with the activations observed by Coull and Nobre [2]. Although VLPFC activation was potentially caused by target detection, we considered the possibility that activation within the VLPFC (Figure 2A–C) may have been caused by another factor. In particular, we were interested in determining whether the right VLPFC activation was at least partially due to spatial selection, as discussed in Rizzuto et al. [32], during the target detection process. To confirm this possibility, we performed SVC based on the main-effect result (Figure 3) analyses, and the results showed that activation in the right VLPFC, was significantly greater for spatial attention tasks when compared with temporal or neutral attention tasks. This finding was inconsistent with a previous study indicating that the bilateral VLPFC is involved in spatial selectivity [32]. However, we note that the findings of Rizzuto et al. were performed in the BA 45/47, while our reported activations were within BA 11/47. Furthermore, our above discussion was based on the fact that we compared the spatial tasks with the control task that our control task was designed to specifically exclude activation related to object cognition (e.g., the target), as well as excluding activation within the VLPFC caused by detection-related processes evoked by hits [30]. Therefore, we believe the most consistent model is that the VLPFC plays a role in the endogenous maintenance of alert states [29], [30] for spatial process. Because we did not observe significant differences in left VLPFC activation between spatial task and the other two tasks, we are less confident that the left VLPFC plays a role in the spatial process during auditory spatial orienting of attention process. This may be explained by the right hemisphere dominance of target detection and spatial selective attention [4].

### Working Memory: Role of DLPFC during Attention Tasks

The DLPFC has been reported to be involved in working memory (WM) [33], [34] and spatial attention shifts [35], [36]. Previous studies [34], [37] on the relationship between WM and attention within the DLPFC hypothesized that WM plays an important role in these processes. WM within the DLPFC not only accepts, stores, and manipulates information but also generates signals that improve the quality of the processed information. In addition, the fact that visual cues can influence the outcome of auditory attention tasks in the rDLPFC [31] could be consistent with the theory of attentional set, with respect to rule and task-relevant information [38].

Although the anatomical ROI-based results (Figure 5B, 5F) within the DLPFC showed averaged BOLD signal-differences between spatial tasks (with respect to the two other tasks), these differences did not survive multiple comparison, while only the significant difference between spatial and neutral taskwithin right DLPFC. We interpret these finding to mean that WM within the DLPFC has an executive function and stores information related to these events [37], [39], [40], with a bias toward spatial information. In addition, we did not find significant differences among the three tasks in further analyses (see the SVC results based on main-effect analysis). The task set used in this study was attentional and aimed to manipulate spatial or temporal attention to a given target. Therefore, we considered that WM is activated to store the rules and the related information, regardless of the specific type of the attention [38].

### Specific Activations during Temporal Task and Neutral Task

Specific activations observed during temporal tasks were in the left SOG and the tegmentum within the midbrain. Interestingly, the SOG was reported to be activated in a study of visual orienting of attention during temporal stimulus [41]. Exogenous and endogenous temporal attention were carried out, and the results showed significant activation within the bilateral extrastriate cortex (BA19) during these processes. In the present study, however, we used an auditory target and observed left SOG activation during the temporal orienting of attention process, which is partially consistent with the previous study. The tegmentum was another region that was specifically activated during temporal tasks, which is in good agreement with a study [42] investigating auditory temporal discrimination. Our finding of activation within the tegmentum can perhaps explain why decrease in activity of this region are observed in Parkinson’s disease patients during presymptomatic stages. Parkinson’s disease patients usually suffer damage to the tegmentum at presymptomatic stages [43] and have declined temporal estimation ability [44].

During neutral tasks, although this process itself might engage attention and orient it along the two spatial locations and the two temporal intervals, it would seem like a superior alternative to one location and one interval [2]. Several regions were observed to be activated (compared with the spatial and temporal tasks), including the bilateral PHG, which is potentially used for implicit priming [45]. The monitoring of targets from bilateral locations caused the SOG to be activated significantly, despite the fact that the target was an auditory stimulus and that eye movement was repressed [20]. As mentioned above, during neutral tasks, attention resources might become divided into spatial and temporal domains, the posterior cerebellum, which is reportedly involved in the prediction of timing of perceptual events within the visual domain [46], was also activated by auditory targets in the present study. On the other hand, posterior cerebellum activation has been considered to be correlated with the non-motor aspects of cognition in the DLPFC (BA46) [47].

### Hazard Function Related Brain Regions

The most prominent difference between a Temporal trial and a Neutral trial is that in the Temporal condition, listeners knew which time interval to pay attention to. However, in the Neutral task, participants did not know the time interval between cue and target. Accordingly, listeners actually need to exert more ‘temporal effort’ during a Neutral trial as compared to a Temporal trial, because they didn’t know which interval was going to contain the target. During Temporal trials, listeners only need to exert their attention on one interval (i.e., the cued interval), whereas in Neutral task (particularly the long interval target Neutral trials), listeners need to exert their attention on two intervals. Accordingly, we considered that activations shown in Figure 8 are related to hazard function [17].

Furthermore, as shown in Figure 9, when participant exerted more ‘temporal effort’ (long interval target trials when the did not know the target onset time) during the neutral, subcortical areas (thalamus and putmen) would be coactivation areas with frontal areas. This finding have a good agreement with previous visual temporal attention study [41]. We hypothesize that the brain temporal cognition network related to auditory stimuli may also include several cortical and subcortical areas that mentioned above.

## Materials and Methods

### Participants

The participants in this study were 16 healthy, right-handed students aged 21–26 years. Written informed consent was obtained prior to participation in the fMRI study. The protocol was approved by the ethics committee of the Peking University Health Science Centre in accordance with the declaration of Helsinki (2008), and all subjects had given their written informed consent.

### Experimental Setup

Visual and auditory stimuli were generated on a computer and presented to the participants via a custom-built, magnet-compatible audio-visual system (at a sound level comfortable to each participant) during MR scanning. In order to attenuate the acoustic noise that accompanies fMRI scanning, shooting ear muffs were used. The auditory stimuli were presented via an air-conductive tube to participants. Presentation 0.61 (http://www.neurobs.com/) was used to generate auditory and visual stimuli.

### Experimental Stimuli and Procedures

During the experimental tasks, the participants were asked to fixate their attention on a central cue and to pay attention to the specific type of cue (spatial or temporal) for one of two spatial directions or one of two temporal interval lengths (totaling four possible outputs). More specifically, during the spatial attention task, the participants were instructed to pay attention to a right or left auditory target based on spatial cue. During the temporal attention task, the participants were instructed to estimate (to the best of their abilities) when the target event would occur, given a visual cue about the length of time to the event. A process of neutral attention (i.e., no induced spatial or temporal attention) was observed to determine nonspecific attention-related activity and used to cancel out basic visual and auditory cognition mediated activity. During the spatial task, participants were told to pay attention to a location of target follow the cue information and respond to the target as quickly as possible when it appeared. During the temporal task, participants were told to anticipate the onset of target by referring to the cue information. Participants were also told to respond to the target as quickly as possible. During the neutral attention task, the cue was uninformative, and the participants were only told to respond to a target as quickly as possible when a target appeared. During the exmperiment, participants have to judge the target location (right or left) and press the reaction key correctly even during the temporal task and neutral task. Correct reaction time was defined when it came in the range of 100–1000 ms after the target onset. Although we do not focus on the results of the neutral attention brain networks in this paper, we want to confirm that the frontoparietal (FPN) network was engaged during variable top-down attention processes [2]; this network in itself engages attention and can orient it along two spatial locations and two temporal intervals and record the activation involved in target selectivity. We considered there are differences in brain activations between temporal and neutral tasks. During the temporal task, participants paid attention to a specific time (early or late), however, they had to pay attention to an early time firstly, then paid attention to the late one if target did not appear in the early time.

**Figure 10:**
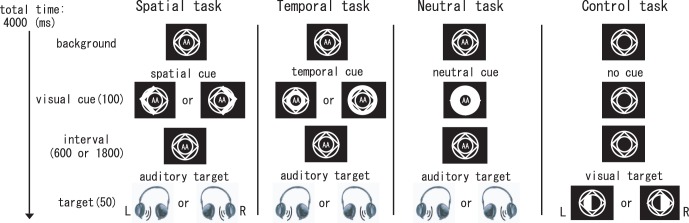
Experimental paradigm and stimuli. A. A flowchart of an individual trial. In this example, the visual spatial cue indicates spatial information but provides no information about the cue–target interval. The cue was lit for 100 ms, and following the appropriate cue–target interval (600 or 1,800 ms), the auditory target was presented for 50 ms. B. Central cues used in the experimental task. The spatial cue was used in the spatial attention tasks. As the stimulus, the right or left half of the cube was lit to provide the subjects information concerning the target location (i.e., right or left). The temporal cue was used in the temporal attention tasks. When the target came within a short cue–target interval, the inner circle was lit; when it came after a long cue–target interval, the outer circle was lit. The neutral cue was used in the control tasks and provided neither spatial nor temporal information. A double ‘A’ in the center of the cue indicated a visual experiment. C. Either the left half or the right half of the inner circle would turn white during the control task, and participants would press the reaction key when the white half round turned to white. RT was not required.

As shown in Figure 10, four separate tasks were used during these studies. Visual cues of spatial or temporal stimuli were used to direct the subject’s attention to one of two possible target locations (left or right) during spatial tasks or one of two time-interval lengths (600 ms or 1,800 ms) during temporal tasks. A third visual cue stimulus, the neutral cue, which provided neither spatial nor temporal information, was used during neutral tasks to prepare participants for target detection.

The spatial, temporal, and neutral cues were used separately during the auditory spatial, temporal, and neutral tasks experiments. Specifically, to display visual cues on the computer monitor, a rhombus located between two concentric circles was displayed in the center of the monitor, as part of the dimmed computer screen background. Visual cues were presented for 100 msec. Spatial attention task cues consisted of causing the left half or the right half of the central rhombus to light up and appear as an arrow (pointing left or right). For the temporal attention tasks, the cue consisted of concentric small and large circles, which lit up and was followed with either a long or short time-interval before the occurrence of the auditory event. For the neutral task, all three geometric shapes lit up (the rhombus and the inner and outer circles) as a non-informative indication of an upcoming stimulus. During all three attention tasks, the interval length between presentation of the cue and the auditory target event was either 600 or 1800 msec, which occurred with equal probability. The auditory target stimulus was a burst of white noise that lasted for 50 ms following the interval after cue-stimulus presentation. The auditory target stimulus included all the frequency from 20–20,000 Hz. An intensity difference between the left and right ear (interaural intensity difference, IID) was used for yielding a lateralised auditory percept. The participants were instructed to press response keys corresponding to either a right (by pressing the “right” key using the middle finger of their right hand) or a left audio stimulus (by pressing the “left” key using the forefinger of their right hand) during all the three attention tasks. We recorded their reaction times (RTs) (the length of time from the onset of the audio target event to the time a key was pressed). The reaction accuracy was also recorded. The participants performed 30 trials under each of the 3 attention conditions. A control task was used as a baseline to cancel out activation caused by detection-related processes evoked by hits [30]. During the control task, the left half or the right half of the inner circle activated (with equal probability) and the participants were asked to press the reaction key once. Control tasks were carried out 30 times in total. All trials duration of attention tasks and control task were 4000 msec. The experimental details were explained to each participant, and a practice/training course was performed before MR scanning. We obtained brain activations of the participants who completed the training task with accuracy over 70%. A block design was used for these experiments, in which the three tasks were randomized in blocks of ten trials, which were carried out three times, for a total of thirty trials. The experiment lasted for a total of eight minutes. The participants were instructed to respond as quickly as possible to the target stimulus.

### fMRI Scanning

Images were acquired using a 3-T Siemens Scanner-vision whole-body MRI system to measure activation with a head coil. The imaging area consisted of 36 functional gradient-echo planar imaging (EPI) axial slices (voxel size = 1.8×1.8×3.9 mm, TR = 4,000 ms, TE = 50 ms, FA = 90°, 128×128 matrix) that were used to obtain T2*-weighted fMRI images in the axial plane. For each participant, we obtained 124 functional volumes.

### Behavioral Data Analysis

RTs were used as behavioral data after excluding invalid trials and the missed trials from each participant. The RT data measured during the fMRI experiments were analyzed using one-way repeated-measure ANOVA with 3 levels (spatial vs temporal vs neutral) and with equal variance assumptions (using the software program SPSS 16.0 for Windows). Bonferroni multiple comparison correction tests (at *p*<0.05) were used for post hoc analyses of pair-wise comparisons.

### fMRI Data Analysis

For the functional image analyses, we first used MRIcro (http://www.cabiatl.com/mricro/) to convert the DICOM files to NIFTI files. The first four functional images were discarded for each run.

Data preprocessing and statistical analyses were performed with the Statistical Parametric Mapping computer package (SPM5; Wellcome Department of Cognitive Neurology, London, UK; http://www.fil.ion.ucl.ac.uk/spm/software/spm5) [48] implemented in MATLAB (The MathWorks). All volumes were realigned spatially to the first volume of the first time series. The movement parameters generated during spatial realignment indicated that all sixteen participants moved less than 2 mm during the course of the trial. Realigned images were spatially normalized using the standard EPI template in the Montreal Neurological Institute (MNI) reference brain coordinate space [49] and resampled into 2×2×2 mm voxels [50]. Normalized images were smoothened using an isotropic 8-mm FWHM (full-width half maximum) Gaussian kernel.

Statistical analyses were performed in two stages of a mixed-effects model. During the first-level of analysis, the BOLD response was modeled as the neural activity convolved with a canonical hemodynamic response function (HRF) [51], which yielded regressors in a general linear model (GLM) for each condition (spatial task vs. control task, temporal task vs. control task, and neutral task vs. control task). The time series in each voxel were high-pass filtered to remove low-frequency noise and scaled (within session) to a grand mean of 128. Nonsphericity of the error covariance was accommodated by an AR(1) (first-order autoregressive) model, in which the temporal autocorrelation was estimated by pooling over the suprathreshold voxels [52].

The contrast (con) images from the first-level analyses from all 16 subjects were then used for the second-level group statistics. To identify the areas of whole brain activation in the spatial task vs. control task, temporal task vs. control task, and neutral task vs. control task samples, a one-sample t-test analysis was carried out for each of the three con images. Only effects passing an uncorrected threshold of *p*<0.0005 and including 1 or more contiguous voxels were interpreted. One-way repeated measure analysis of variance (ANOVA) was used to examine the activation differences between the 3 conditions. We used ANOVA with repeated measures because the 3 conditions were the same for each subject, and there was also a common baseline for the 3 conditions for each subject. To examine the conditional differences over the ROIs, SVC procedures implemented in SPM were applied. Based on previously reported findings, we defined Six SVC masks using the WFUPickAtlas software toolbox (http://www.fmri.wfubmc.edu/cms/software#PickAtlas) in SPM. These 6 masks were Frontal_Mid_R for right DLPFC, Frontal_Mid_Orb_R for right VLPFC, Frontal_Mid_L for left DLPFC, Frontal_Mid_Orb_L for left VLPFC, R_Supramarginal_Gyrus for right inferior parietal lobe, and R_Supramarginal_Gyrus for left inferior parietal cortex. In addition, two masks, left Brodmann area (BA) 21 and right BA 21) were also defined by the WFU PickAtlas. We used SVC with family-wise error controlled at *p*<0.05 for the analysis of the following: (spatial task vs. control task) vs. (neutral task vs. control task), and (spatial task vs. control task) vs. (temporal task vs. control task).

To further evaluate the significant differences in regional signal change between the three conditions, the anatomical ROIs noted above were used to extract the averaged data out of the first level individual subject statistical analyses. For each ROI, there was a total of 48 measurements (3 conditions for each of the 16 participants). These data were then used in repeated measure ANOVA tests (SPSS 16.0 for Windows) with an equal variance assumption. Measurements of non-parametric were used for the post hoc analyses to examine the statistical significance of each ROI.

### Conclusions

In conclusion, during the auditory spatial attention process, we observed activity in the dorsal FPN, including the bilateral IPL and the bilateral FEF, as well as in the PFC, including the right middle PFC (BA6), the bilateral DLPFC and the VLPFC. When compared with the activity during the temporal and neutral attention processes, significant differences in activation were found in the bilateral IPL and the right VLPFC. However, bilateral DLPFC activations were observed during the three auditory attention processes without significant differences. With respect to particular activations, we found that the tegmentum within the midbrain activity was associated with the auditory temporal process, and activity was also observed in several regions related to auditory/spatial-temporal processes, such as the bilateral PHG, the SOG and the left posterior cerebellum bilateral FEF, as well as the bilateral insula, subcortical areas (thalamus and putamen). We believe that our findings on multiple task-specific regions are likely to be a useful reference for further studies, such as connectivity studies using dynamic causal modeling (DCM).
